# In situ tagging technique for fishes provides insight into growth and movement of invasive lionfish

**DOI:** 10.1002/ece3.1171

**Published:** 2014-09-10

**Authors:** John L Akins, James A Morris, Stephanie J Green

**Affiliations:** 1 Reef Environmental Education Foundation98300 Overseas Hwy, Key Largo, Florida, 33037; 2 NOAA National Ocean Service, National Centers for Coastal Ocean Science, Center for Coastal Fisheries and Habitat Research101 Pivers Island Rd., Beaufort, North Carolina, 28516; 3 Department of Biological Sciences, Simon Fraser University8888 University Drive, Burnaby, British Columbia, V5A 1S6, Canada; 3 Department of Integrative Biology, Oregon State University3029 Cordley Hall, Corvallis, Oregon, 97331-2914

**Keywords:** Animal health, animal movement, fish behavior, invasive species, mark-recapture, *Pterois volitans*, red lionfish, site fidelity, tagging method

## Abstract

Information on fish movement and growth is primarily obtained through the marking and tracking of individuals with external tags, which are usually affixed to anesthetized individuals at the surface. However, the quantity and quality of data obtained by this method is often limited by small sample sizes owing to the time associated with the tagging process, high rates of tagging-related mortality, and displacement of tagged individuals from the initial capture location. To address these issues, we describe a technique for applying external streamer and dart tags in situ*,* which uses SCUBA divers to capture and tag individual fish on the sea floor without the use of anesthetic. We demonstrate this method for Indo-Pacific lionfish (*Pterois volitans/P. mile*s*),* species which are particularly vulnerable to barotrauma when transported to and handled at the surface. To test our method, we tagged 161 individuals inhabiting 26 coral reef locations in the Bahamas over a period of 3 years. Our method resulted in no instances of barotrauma, reduced handling and recovery time, and minimal post-tagging release displacement compared with conventional *ex situ* tag application. Opportunistic resighting and recapture of tagged individuals reveals that lionfish exhibit highly variable site fidelity, movement patterns, and growth rates on invaded coral reef habitats. In total, 24% of lionfish were resighted between 29 and 188 days after tagging. Of these, 90% were located at the site of capture, while the remaining individuals were resighted between 200 m and 1.1 km from initial site of capture over 29 days later. In situ growth rates ranged between 0.1 and 0.6 mm/day. While individuals tagged with streamer tags posted slower growth rates with increasing size, as expected, there was no relationship between growth rate and fish size for individuals marked with dart tags, potentially because of large effects of tag presence on the activities of small bodied lionfish (i.e., <150 mm), where the tag was up to 7.6% of the lionfish's mass. Our study offers a novel in situ tagging technique that can be used to provide critical information on fish site fidelity, movement patterns, and growth in cases where *ex situ* tagging is not feasible.

## Introduction

Information on animal movement patterns and growth rates are essential inputs into ecological models that guide population management activities. Among the most common approaches for obtaining these data is the application of external (i.e., visual) tags, and subsequent resighting or recapture of marked individuals (Turchin 1998). In fishes captured at depth and tagged at the surface, tagging often has serious effects on the health and behavior of captured specimens (Parrish and Moffit 1992). Of primary concern are the effects of barotrauma and surface holding time on health and survival (Nichol and Chilton 2006; Gravel and Cooke 2008; Jarvis and Lowe 2008; Rudershausen et al. 2014) and spatial displacement following release on behavior and movement patterns (Parker et al. 2006; Schreer et al. 2009). Negative effects of chemical anesthesia and lengthy recovery time during the tagging process are also documented (Anderson et al. 1997; US FDA, 2011). In addition, substantial personnel and logistic resources are required to facilitate the lengthy tagging and recovery process (Hammer and Lee Blankenship 2001). However, methods to mark individuals in situ, such as implanting internal tags via pole hooking (Irigoyen and Venerus 2008) or surgery, (Parker et al. 1990; Starr et al. 2000; Lindholm et al. 2005), or external dart tag application through spearing (Adkison et al. 1995), can help to address some of these issues, depending on the species, its environment and logistical considerations.

Here we describe in detail an alternative in situ external tagging method that can be used to collect information on growth rates and movement patterns, and has the potential to address all of these concerns simultaneously. We use this method to collect movement and growth information for Indo-Pacific lionfish (*Pterois volitans* and *P. miles*), an invasive species for which these data are urgently needed to inform control strategies, but for which physiological and ecological constraints greatly limit the utility of conventional *ex situ* tag application (Akins 2012). We report the results of our tagging efforts in terms of fish handling time and health upon release, and compare logistic considerations of this technique with those for standard *ex situ* tagging. Finally, we interpret data on movement and growth gathered from resighting tagged lionfish in the context of designing effective control programs for the invasion. Our ultimate goal is to produce an in situ tagging method that can be used to mark and track fishes, in instances where *ex situ* external tag application is not feasible.

### Focal species

Lionfish were first sighted off the coast of South Florida in 1985, and have since spread rapidly throughout the southeast United States, the Caribbean Sea, and Gulf of Mexico (Morris and Green 2012). As invasive predators, lionfish are having significant impacts to reef fish community structure and biodiversity as a result of direct predation on a wide array of native fish and crustacean species (Morris and Akins 2009; Green et al. 2012; Côté et al. 2013). Removal of lionfish by humans, via nets and spears, is currently the primary method used to suppress their populations (Akins 2012). An understanding of lionfish site fidelity, movement patterns and growth rates on invaded marine habitats will assist managers with developing control plans.

The physiology and ecology of Scorpaenid fishes, such as lionfish, greatly limit the utility of conventional *ex situ* tagging procedures for gathering growth and movement information. In particular, Scorpaenids, a physoclistous teleost, suffer high rates of mortality due to barotrauma associated with surfacing from depths as little as 18 m, and the risk of mortality increases rapidly with handling time at the surface (Parker et al. 2006; Jarvis and Lowe 2008). These effects appear to be manifested in lionfish as well, with preliminary *ex situ* tagging efforts where specimens were brought up from depths of 3–6 m and anesthetized with MS222 for the application of external Floy tags resulting in 100% mortality of collected specimens (N. Smith, pers. comm.). In addition, lionfish are reef-associated predators that utilize specific habitats for hunting and sheltering (Green et al. 2011). Thus, limited control over release location during *ex situ* tagging may result in lionfish being translocated to potentially undesirable habitat, with potentially large effects on their behavioral ecology, movement patterns, survival, and growth rates.

## Methods

### Sampling locations

**Figure 1:**
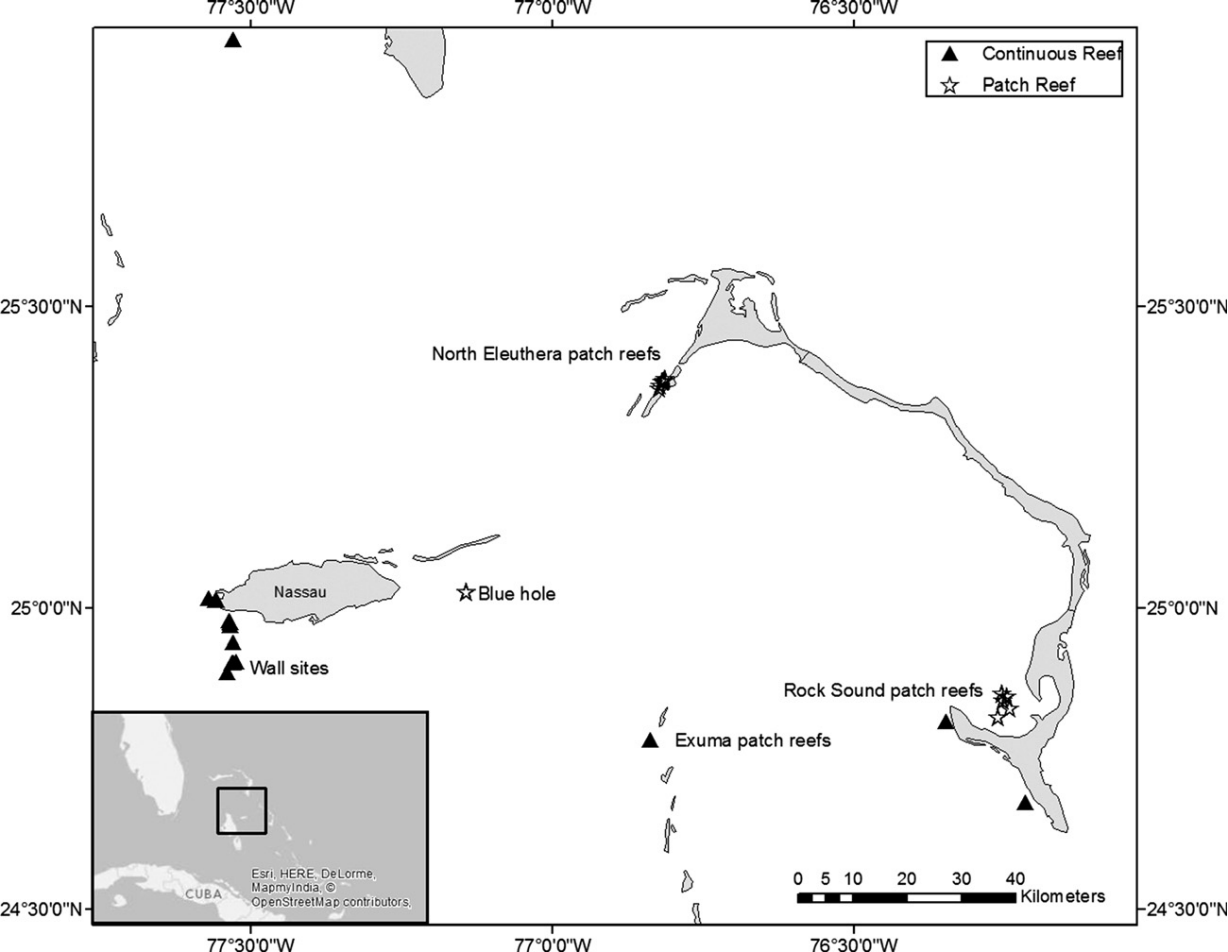
Location of fish capture and in situ tagging in the north-central Bahamian archipelago. Triangles indicate continuous coral reef habitats, stars indicate patch reef habitats.

Between November 2007 and June 2010, we collected and tagged 161 lionfish at 26 locations off three islands in the north-central Bahamian archipelago. Sites represented a range of depths on either continuous or patch reef habitats, and were selected due to the abundance of lionfish and frequency of visitation by local dive operators (Fig.1). Specifically, we tagged lionfish at 11 locations along a continuous reef system adjacent to deep water walls of the Tongue of the Ocean, off southwest New Providence Island, at depths of 10–20 m; 14 shallow patch reefs off the Exumas and Eleuthera Island featuring isolated coral heads at depths of 2–10 m surrounded by sand and seagrass, and separated by approximately 200–400 m from other similar coral structures; and patch reefs surrounding a blue hole on the Bahama Bank, consisting of sheer wall structure to a depth of 51 m, composed of a remnant flooded cavern, with coral features dominated by *Montastrea* spp. and *Diploria* spp. colonies circling the top of the cavern at a depth of 12 m, surrounded by seagrass meadows (Fig.1). All tagging areas were part of ongoing lionfish research by the authors and subject to opportunistic visitation and recapture events.

### Tagging procedure

The tagging process can be characterized in five phases, all of which took place in situ. Images (Figs.5) and video (Videos S1 and S2) footage of each step are referenced in the text below.

**Figure 2:**
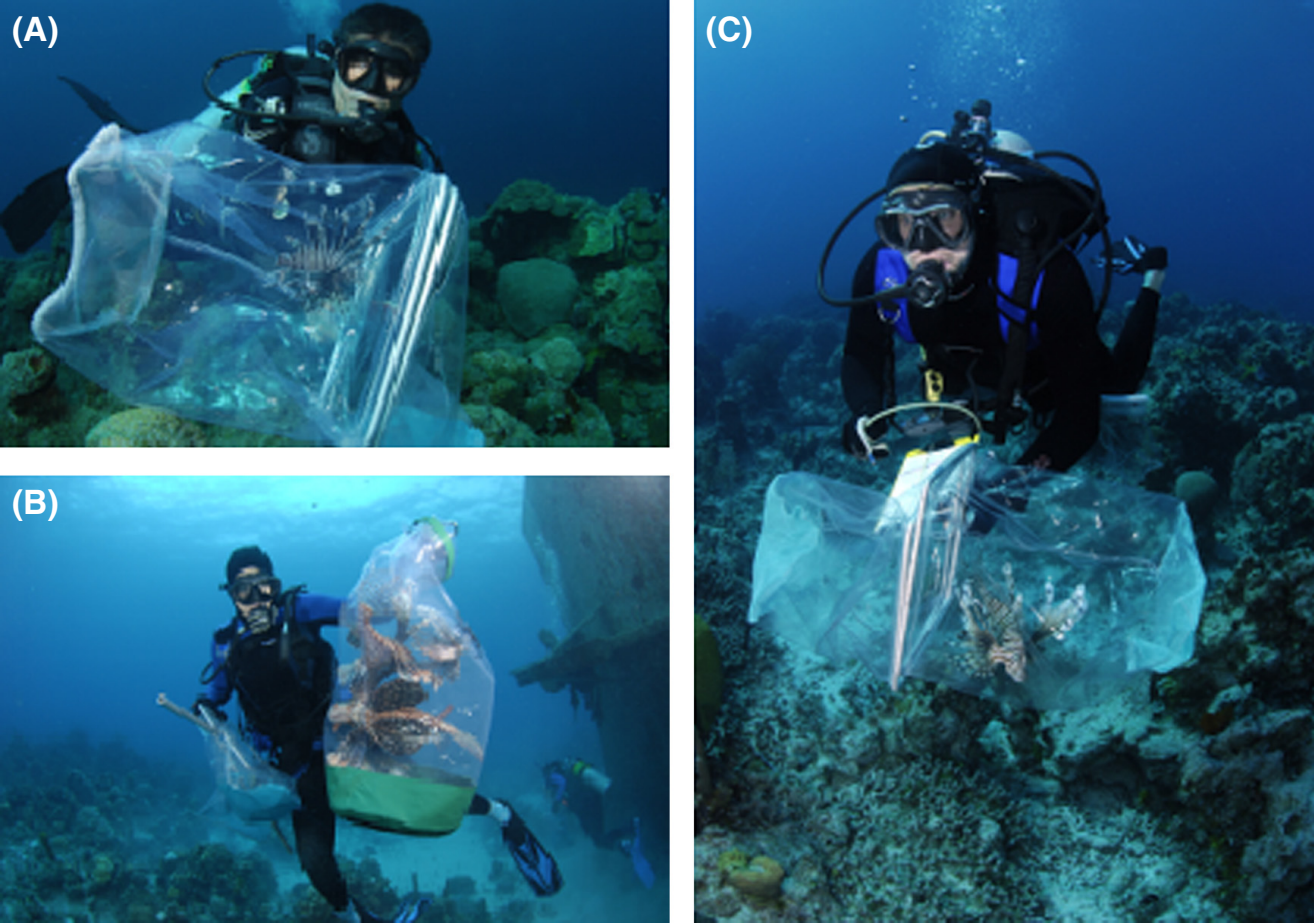
In preparation for in situ tagging, fish are (A) collected using hand nets and then transported to the tagging station using either (B) a clear dry bag, or (C) the two hand nets.

**Figure 3:**
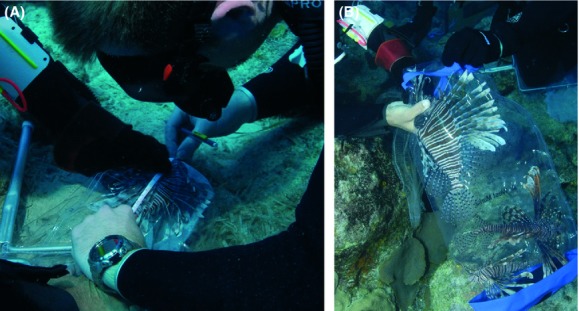
Prior to tag placement, total and standard lengths are measured for each fish by either (A) measuring the specimen while in the collection net or (B) removing them from the collection bag.

**Figure 4:**
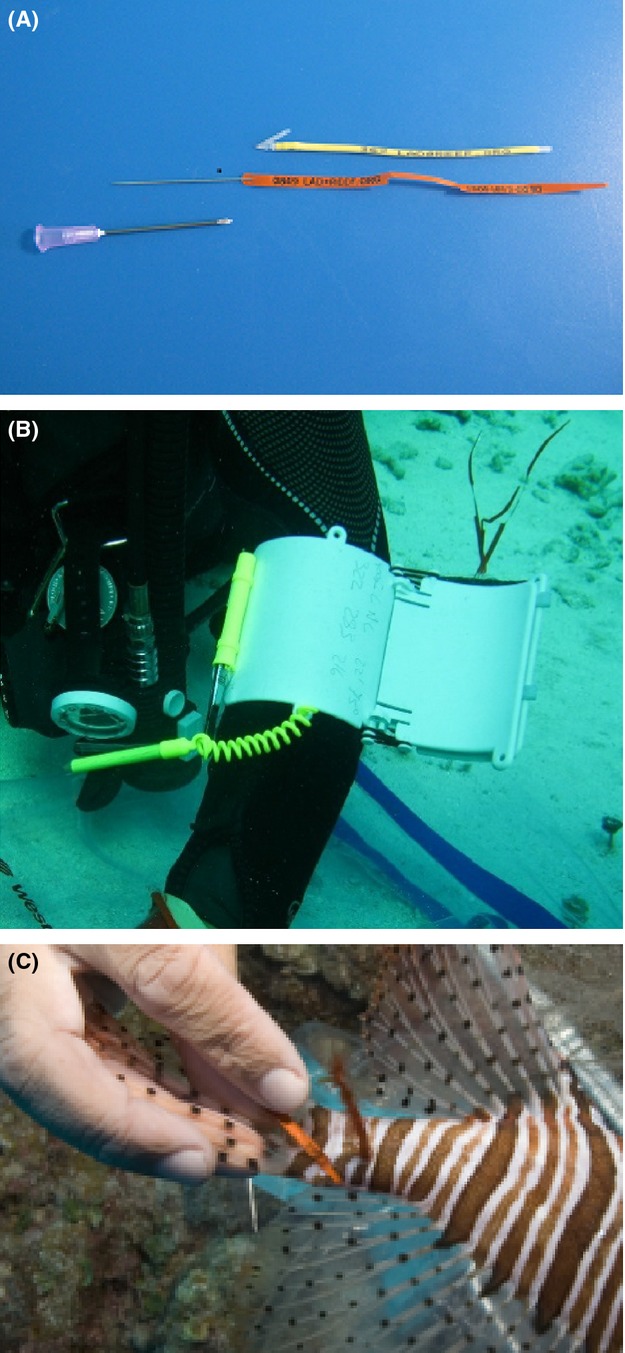
Tagging equipment. (A) From top to bottom, a Floy dart tag, Floy streamer tag and sample tagging IV needle. (B) Wrist slate with tags embedded in foam strip. (C) Streamer tag #064, recovered 73 days post tagging.

**Figure 5:**
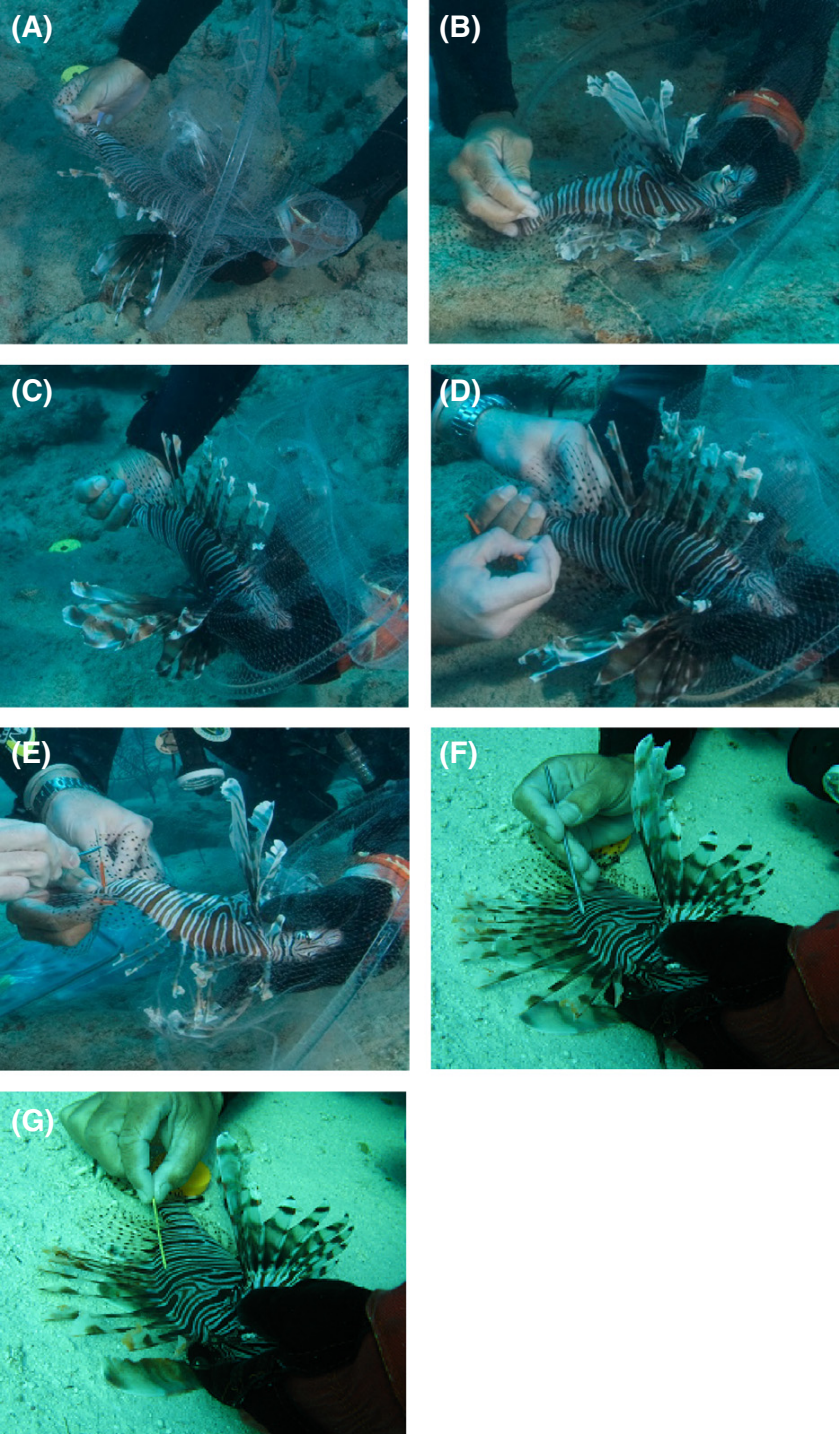
In situ tagging process. (A) Location of IV needle insertion. (B) Positioning of fish against bottom to facilitate insertion of IV needle. (C) Fish repositioned with team members facing each other. (D) Insertion of tagging needle into IV needle. (E) Positioning of tag midway through caudal peduncle. (F) Positioning of dart tag showing insertion tool. (G) Inserted dart tag.

#### Fish collections

Fish were collected live with hand nets using the capture techniques described by Akins (2012). Capture via hand net tends to work well for slow moving demersal fishes that inhabit structurally complex habitat, such as lionfish; however, alternative capture methods such as trapping and in situ hook and line captures could be employed for other species. All captures were conducted using SCUBA by trained and experienced personnel working in teams of two or three. At each site, lionfish were located by a search team during a roving survey of the habitat within a 50 m radius of the dive support vessel. Once located, the search team worked together using clear vinyl or monofilament mesh hand nets to capture each lionfish individually (Fig.2A and Video S1).

#### Transport to tagging station

Following capture, fish were either transferred to a large clear vinyl containment bag or secured between two collection nets for transport to the tagging station (Fig.2B and C; Video S2 00:01). Tagging stations were located on the bottom near the dive support vessel. Care was taken to locate stations at a depth similar to capture depths in order to eliminate barotrauma, as well as in areas of sand or rubble to avoid disturbance of living benthos. The tagging station was manned by a team of two divers who were responsible for taking measurements of and applying external tags to each fish. Each tagging team was supplied with tags, forceps, measuring tapes, and puncture resistant gloves and positioned themselves facing each other approximately 1 m apart. In most cases, the two designated tagging divers entered the water following the first collecting team to allow time for initial captures. Once in possession of the specimen, the tagging team worked together to collect measurements and apply an external tag to each lionfish.

#### Measurement

Prior to tag application, the condition (i.e., appearance and behavior) of each fish was visually assessed to ensure normal appearance and behavior following collection. Any injured or otherwise abnormally appearing fish would have been rejected from the tagging pool. Next, the tagging team obtained length measurements (standard and total lengths) for each fish using one of two methods (Fig.3A–B). The first involved collapsing the clear vinyl net or bag down on top of the fish and positioning the fish laterally to allow visual access to the body of the fish including head and caudal fin (Fig.3A). When handling lionfish, care was taken to use puncture resistant gloves and caution was exercised when collapsing the bag near venomous spines. Standard and total length measurements of the fish were taken by use of small metric tape measure. Alternatively, if multiple fish were collected and presented in a single bag at the tagging station, fish were individually removed from collection bag for measuring (Fig.3B; Video S2 00:04). This procedure involved one team member (from here on called the “holder”) collapsing the bag on top of the fish with one hand to prevent movement and using the other hand, reaching into the bag and firmly grasping the fish from the front of the head. Once grasped, the fish was removed from the bag and positioned laterally on the bottom. The caudal fin was then grasped firmly by the holder and the fish held in place. Once in position, the fish typically became immobile. The second member of the tagging team (from here on called the “tagger”) then used a flexible tape measure to obtain length measurements. To validate accuracy of in-water measurements, three individual fish were measured on the bottom following collection, then taken to the surface and remeasured during same day dissections; for all fish, length measurements taken underwater differed by <1% from topside length measurements.

#### Tag application

We applied one of two external tag types to each fish: a Floy® dart (FT-2-94) or a Floy® streamer (FTSL-73) tag (Floy Tag and Mfg, Inc., Seattle, WA. USA) (Fig.4A). Streamer tags consisted of a 102 mm polyethylene strip, weighing 0.02 g, with a glue-attached needle. Dart tags consisted of nylon barbed tips with shrink wrapped polyolefin tubing cut to 87 mm in length and weighing 0.27 g. All tags were color coded and imprinted with three digit serial numbers and phone and email contact information (Fig.4A). Tags and other tagging instruments were held in place by insertion into a closed cell foam pad attached to a three tiered wrist slate (Fig.4B).

To reduce the rate of streamer tag breakage during insertion (i.e., primarily, tag dissociation from the tagging needle), an 18 gauge 1½″ intravenous (IV) needle was used as a guide. While the holder focused on maintaining immobility and position of the fish, the tagger inserted the IV needle laterally through the dorsal musculature approximately one-quarter the distance anteriorly from the caudal peduncle (Fig.5A). To initiate the insertion, the tagger either angled the leading edge of the IV needle underneath a scale edge or used the tip of the IV needle to clear a small area of scales at the area of insertion to minimize scale interference, then straightened the needle to a position perpendicular to the peduncle. To facilitate needle passage fully through the caudal peduncle, the fish was held onto the bottom to provide a background for increased pressure during insertion (Fig.5B; Video S2 00:22). Following IV needle insertion, the fish was repositioned with the needle opening toward the tagger (Fig.5C; Video S2 00:36). The streamer tag was then pulled from the tagging slate and the serial number recorded. The tagging needle was then inserted into the opening of the IV needle, the fish was repositioned with the insertion side again facing the tagger and the entire needle assembly pulled back through the insertion side (Fig.5D; Video S2 01:00). After removing the IV needle, the tagger gripped the tagging needle with forceps and pulled the streamer tag half way through the caudal peduncle, leaving half of the streamer protruding along either side of the peduncle (Fig.5E, Video S2 01:10). To complete the tagging procedure, the tagging needle was detached from the plastic streamer (Video S2 01:20).

Dart tag insertion followed the same basic team handling procedure used for streamer tags, differing in that dart tags were inserted using a Floy® applicator tool (FT-2) and inserted at an anterior angle approximately 2–5 mm below the 4th or 5th dorsal spine (Fig.5F). Following insertion, the tool was removed and the tag lightly tugged to set the dart behind the interneural bones. The remaining exterior portion of the tag was angled posteriorly and along the body of the fish (Fig.5G).

#### Fish recovery and release

Following tagging, all lionfish were placed into collection bags or nets and returned to the reef within 5 m of the capture location. Following release, each fish was observed to note postcapture behavior. Fish recovery was categorized based on the time to resumption of normal behavior and the presence of any capture-related injuries (i.e., lost scales and fin damage; minimal scale loss = good, moderate scale loss = fair, scale loss, equilibrium loss and/or fin damage = poor).

### Resighting and recapture

Tagging locations and adjacent reef sites were revisited opportunistically throughout the study period and the presence of tagged fish noted. When possible, tagged specimens were captured and remeasured or removed from the water. Recaptured lionfish were euthanized using approximately 45 mL of a 10% mixture of eugenol (clove oil and alcohol) diluted in 3–5 gallons of seawater and then placed on ice following the protocols of Green et al. (2012). To assess growth between tagging and recapture, total and standard length measurements were again taken from each specimen.

### Calculating growth and movement rates

We calculated growth rate (in mm/day) as the difference in (TL) divided by the number of days between initial tagging and recapture. Because growth rate decreases with increasing fish size in a linear fashion (FAO 1998), we estimated the slope and intercept of the relationship between lionfish size at time of tagging (TL in mm) and growth rate (mm/day) from linear regression models constructed using the function lm() in the statistical software language R (R Development Core Team 2012). We hypothesized that tag type might affect lionfish growth rate, so we analyzed the relationship separately for individuals tagged with either Floy dart or streamer tags.

## Results

We tagged a total of 161 lionfish varying in size from 71 to 330 mm total length (mean ± standard deviation = 205 ± 57 mm). Of these, 26 were tagged with dart tags (only at Exuma and Blue Hole patch sites) and 135 with streamer tags (at all sites). All lionfish survived the tagging process and exhibited full recovery in <1 min from release. No lionfish exhibited handling-related injuries, and no individuals were in “poor” condition postrelease. The average time required to tag an individual specimen (from transfer to the tagging station, to release at the collection location) was three minutes, with a maximum of 16 fish tagged during one 46 minute dive. All lionfish were successfully returned to the exact initial capture location on each coral reef.

In total, we recovered or resighted 24% of tagged lionfish between 8 and 188 days after tagging [36 ± 48 days; mean ± SD; *N* = 41; (14 recoveries and 27 sightings)] and observed no instances of infection at the tagging site on all fish. The size of lionfish resighted or recaptured did not differ significantly from those that were not seen again (two-tailed *T*-test; *t* = 1.2992, df = 68.494, *P* = 0.1982). Of the 89 lionfish tagged on continuous coral reefs off New Providence, all individuals that were relocated (either sighted or recaptured; *n* = 30, 34%) were found at their original capture site between 8 and 188 days after tagging, and no tagged fish from these areas were sighted or recovered during searches at adjacent reef sites. Of the 72 lionfish tagged on shallow patch reefs off Eleuthera, Exuma, and Bahamas Bank, 15% (*n* = 11) were relocated. Relocated fishes displayed a variety of movement patterns on patch reefs; 64% (*n* = 7) of individuals were located at the initial tagging site, with 2 fish documented at the same patch reef 188 days after tagging (Table1). However, 37% (*n* = 4) of individuals moved to patch reefs ranging from 215 to 1102 m from their original capture location 29 days after tagging. Most fish were resighted or recaptured only once, though two fish were recaptured twice. All fish were removed following recapture for dissection. No additional sightings of tagged fish occurred beyond 188 days.

**Table 1:** Growth assessment of lionfish tagged in situ with Floy streamer and dart tags

Starting total length (mm)	Days elapsed	Total growth (mm)	Growth day (mm/day)	% Increase per day	Annual growth (mm/year)	Tag type	Habitat type	Depth (m)
112	188	107	0.6	0.51	208	Streamer	Patch	3
118*	48	7	0.2	0.12	53	Dart	Patch	11
118*	37	8	0.1	0.18	79	Dart	Patch	11
135	188	94	0.5	0.37	183	Streamer	Patch	3
140	73	37	0.5	0.36	185	Streamer	Continuous	23
150*	48	25	0.5	0.35	190	Dart	Patch	11
150*	37	20	0.5	0.36	197	Dart	Patch	11
153	37	11	0.3	0.19	109	Dart	Patch	11
183	37	7	0.2	0.10	69	Dart	Patch	11
214	178	86	0.5	0.23	176	Streamer	Continuous	14
235	29	10	0.3	0.15	126	Dart	Continuous	8
241	73	19	0.3	0.11	95	Streamer	Continuous	15
275†	29	8	0.3	0.10	101	Dart	Continuous	8
317	73	12	0.2	0.05	60	Streamer	Continuous	15

* Same fish recaptured twice on different dates. Days elapsed represent total days from initial tagging.

† Tag had pulled out but fish was recaptured and retagged 1 month later. Fish was identified by scarring at tagging site.

**Figure 6:**
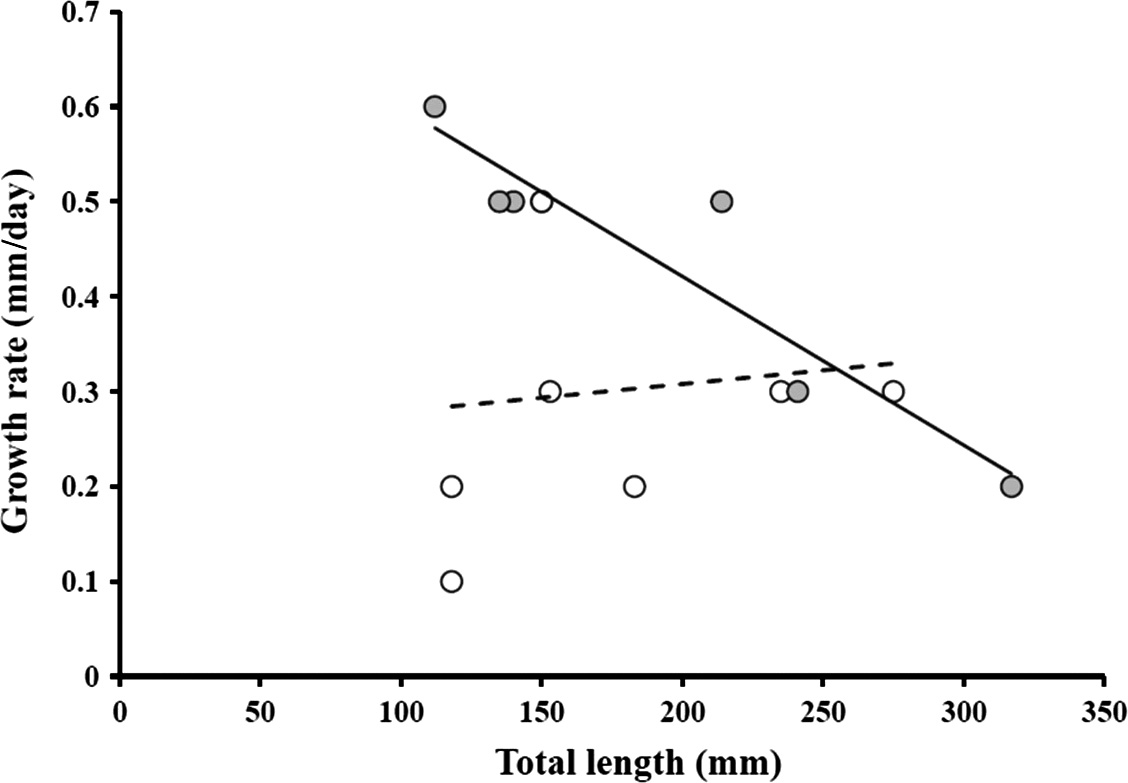
Growth rates of lionfish recaptured following in situ tagging on Bahamian coral reefs. Grey points represent fish tagged with Floy streamer tags, with the solid line representing the relationship between growth rate (GR) and lionfish size (TL) as GR = −0.367 * ln(TL) = 2.302 (*R*^2^ = 0.83, *n* = 6). Open points represent fish tagged with Floy dart tags, with the dashed line representing the relationship between growth rate (GR) and lionfish size (TL) as GR = 0.00472 * ln(TL) + 0.0982 (*R*^2^ = 0.0078, *n* = 8). Lionfish size is TL at time of tagging.

Lionfish growth, measured as the increase in standard length (mm) divided by days between initial and final measurements, ranged between 0.1 and 0.6 mm/day (Fig6). For fish tagged with Floy streamer tags, larger fish exhibited slower growth (Fig.6; GR = 0.77–0.002*(TL); *F*(1,4) = 24.54; *R*^2^ = 0.82; *P* > 0.01; *N* = 6). However, growth rate was not significantly related to fish size for individuals tagged with dart tags (Fig.6; GR = 0.25 + 0.0002*(TL); *F*(1,6) = 0.079; *R*^2^ = 0; *P* = 0.78; *N* = 8).

Tag performance was assessed from the condition of recaptured individuals. Fish recaptured >37 days showed fibrous tissue growth on the peduncle at the streamer tagging site. We only observed one fish with scarring at the tagging site, but no tag; an individual was resighted after 29 days with minor scaring in the dorsal musculature, presumably at the site of dart tag application. Streamer tags from fish resighted after 29 days displayed minimal fouling, and tags were readable both visually and from photographs taken from a distance of approximately 1–2 meters. Streamer tags from fish recaptured after 73 days displayed variable fouling (i.e., algal cover ranging from ˜20% to 60%), and as a result ˜1/2 the tags could be read without clearing off algal growth, which was easily accomplished by a gentle finger scrape. Streamer tags from fish collected after 188 days exhibited heavy fowling, and tags were unreadable without removing algal growth.

## Discussion

The method we develop here to apply external tags to fishes in situ could result in at least four advantages over traditional methods that involve transporting similar specimens to the surface. First, the in situ method we present results in a relatively short specimen handling time. Given that the duration of captivity dramatically increases the magnitude of physiological stress and decreases performance of fishes (Strange and Cech 1992; Schreck 2000), the reduction in handling and recovery time afforded by our method in comparison with those of commonly used surface methodologies (up to 21-d, US FDA, 2011) is likely to increase the wellbeing of specimens following release. Second, our method eliminates the risk of barotrauma within tagged specimens. While surface tagging requires the ascent and descent of collected specimens, and the resulting likelihood of barotrauma injuries associated with swim bladder expansion increases with depth (Parker et al. 2006), specimens tagged via our in situ method are maintained at their capture depth, eliminating health issues associated with pressure change. Third, tagged fishes are free from the effects of anesthesia. At the surface, specimens are typically anesthetized using MS-222, quinaldine, or eugenol, with raw water typically circulated over the gills of the fish for aeration while measurements are taken and tagging initiated. Following successful completion of the tagging procedure, the fish must be revived prior to release. This revival often involves removal from the site for a period of hours or days to ensure full recovery from anesthesia and ascent. For some anesthesia, governing regulations may prohibit the release of potentially edible fish or require lengthy prerelease containment (21 days for MS-222), (US FDA, 2011). Finally, our in situ method releases tagged fish at the same location and same relative time they were captured. During traditional tagging efforts, once recovery is deemed successful, the fish is placed back into the water and released using various methods including surface release, weighted release lines, diver release or open door cage release (Theberg and Parker 2005). Placement on the reef is often randomly associated with position of the vessel and may not be near the capture location or depth. Time of day of release as well as elapsed time could also affect ecological relationships and competitive interactions with other reef inhabitants and conspecifics including displacement from key sheltering or feeding locations.

Short handling time and lack of anesthetic used during our in situ tagging process could also reduce logistical difficulties. For example, in many remote field locations, the disposal of chemical such as MS222, which is harmful to the environment, is not practical. Moreover, our in situ tagging method may reduce the costs of data collection through shorter time requirements of personnel and surface support, and elimination of facilities for holding captured specimens.

Identifying the most appropriate method for tagging fishes depends on a combination of physiological, environmental, and logistical variables. For example, in-water collection and handling of specimens, as conducted in our method, may not be practical for larger pelagic or fast moving fishes, which may be more easily captured via hook and line, and handled near the surface. Also, the logistical support needed for SCUBA is not often available in remote locations, and the cost of such support may be prohibitive depending on available resources. Finally, snorkeling or other capture methods may allow surface tagging without barotrauma or displacement when working with fishes encountered in shallow waters (i.e., <5 m; e.g., Jud and Layman 2012). Thus, our method is likely most appropriate for demersal fishes at depths where barotrauma is likely to occur upon surfacing.

Our study also demonstrates that this method can be used to gain insights into lionfish growth and movement on invaded reefs. To date, growth rates for invasive lionfish in marine systems have been assessed through otolith analysis (Potts et al. 2010) and during laboratory bioenergetics studies (Cerino et al. 2013). The growth data gathered during this in situ tagging trial approximates estimates published for smaller specimens of 67–75 mm total length from the Caribbean (Albins 2013), and 66–256 mm total length in estuary systems in Florida (Jud and Layman 2012). Interestingly, one lionfish tagged with a dart tag, smaller than conspecifics tagged at the same site with the same methods, was recaptured twice and exhibited slower growth rates than the larger specimens, potentially indicating effects of this tag type on fish health and development.

When considering differences in tag type and potential effects on growth, two factors may warrant consideration. First, mass of dart tags is more than ten times that of streamer tags. When considering relationship of tag mass to the mass of the smallest fish tagged in this study, a dart tag represents 7.6% of mass, while a streamer tag represents 0.6%. Another noticeable difference between the tag types is the flexibility of the tags and subsequent position of the tag along the body of the fish. Streamer tags are very flexible and tend to lay flat along the caudal peduncle and tail fin with only half the tag (<50 mm) protruding on either side of the fish. Dart tags are much less flexible and the entire 102 mm tag protrudes at an acute angle from a single side of the fish. The increased mass and obtrusive orientation of dart tags relative to streamer tags, especially among smaller fish, could present significant obstacles to movement and interference to behaviors affecting growth. These factors warrant further investigation to determine tag type effects.

Of the 161 lionfish tagged for our study, 76% were not sighted again during subsequent visits to the study sites and adjacent reef habitats. Tag return rates are highly variable across studies of fish movement and are dependent upon many factors (Pollock et al. 2001). The 24% resighting we obtained through opportunistic site visits should be interpreted with caution, though this return rate is comparable to other mark and recapture efforts. Failure to relocate tagged fish may be explained by a number of factors, including tag loss, tagging induced or natural mortality, poor detection of fish within each site, unreported removals or movement to areas outside the study locations. Tank trials with streamer tags indicate extremely high retention rates and no instances of infection for lionfish (i.e., 97% retention after 10 weeks held in seawater; *N* = 30 lionfish), however similar trials with dart and disk tags resulted in rapid shedding and infection (J. A. Morris, unpubl. data). Moreover, the 100% recovery rate following release from tagging in this study, and the lack of lionfish predators on invaded habitats suggest that tagging-induced and natural mortality rates for this species are relatively low. With many of the New Providence coral reef sites (where 56% of fish were tagged) in close proximity to deep reef wall and ocean trench habitats, it is likely that lionfish moved to depths beyond the limits of detection by divers. Alternatively, lionfish could have moved away from the wall into shallow seagrass and patch reef habitat also outside the study area. Of the documented movement of the three fish in North Eleuthera, one moved south while the other two fish moved north and all three fish remained in similar depth and habit between tagging and resighting. Net movement of lionfish (1239 m) was greater in the northern direction toward other study sites, although no other movement was detected in the three northernmost sites.

Ecological models that guide fish population management often require movement and growth rate inputs. For lionfish, determining patterns and drivers of fish movement across invaded habitats is of critical importance for estimating population control measures, but remains among the least rigorously assessed aspect of their ecology. Tagging and relocating individuals via our method confirms anecdotal reports that some lionfish exhibit high site fidelity and minimal movement over extended periods, but most importantly, highlights that movement is variable between individuals and across habitat types and conditions. Our in situ tagging method is of value for quantifying the conditions under which site fidelity occurs, and ultimately where and when early detection and rapid response removal efforts, as well as more lengthy local control programs, are likely to be successful.

Traditional methods of *ex situ* tag application are often traumatic for collected specimens, require lengthy ascents and recovery times and can place fish significant distances from the initial capture location. Our unique method of affixing external tags underwater on SCUBA can be an effective and efficient method of gathering information on fish movement and natural mortality while minimizing trauma and displacement.

## Supporting Information

Video S1 Collecting and handling fish specimens in preparation for in situ tagging.

Video S2 Applying external (visual) tags in situ.
